# P15 From pandemic to policy: leveraging COVID-19 lessons to strengthen antimicrobial stewardship

**DOI:** 10.1093/jacamr/dlaf118.022

**Published:** 2025-07-14

**Authors:** Rasha Abdelsalam Elshenawy

**Affiliations:** School of Health, Medicine and Life Sciences, University of Hertfordshire, UK

## Abstract

**Background:**

The COVID-19 pandemic disrupted healthcare services, straining antimicrobial stewardship (AMS) efforts. Uncertainty about bacterial co-infections led to widespread empirical antibiotic use, heightening antimicrobial resistance (AMR) concerns. Fewer than 10% of COVID-19 patients had confirmed bacterial infections, yet over 70% received antibiotics.^1^ Translating research into evidence-based policies is crucial for strengthening AMS, promoting health system resilience, and ensuring preparedness for future global health emergencies.^2,3^

**Objectives:**

To evaluate hospital-based AMS practices during COVID-19 and translate insights into a policy brief highlighting the pandemic’s impact, lessons learned, and recommendations for resilient, sustainable AMS and global AMR policy improvements.

**Methods:**

A comprehensive evaluation of AMS practices during COVID-19 included three studies. A literature review examined global AMS implementation over 20 years. Antibiotic prescribing patterns in 640 patients across two NHS Trust hospitals (2019–2020) were analysed. A survey of 240 healthcare professionals explored knowledge, attitudes, and perceptions of antibiotic prescribing, AMR, and stewardship.^4^ This was further enriched by reviewing institutional guidelines, national and global policy responses—such as WHO advisories on antibiotic use and AMR surveillance—and key governmental and published reports.

**Results:**

The findings informed the development of a policy brief^5^ addressing three key areas: the impact of COVID-19 on AMS, critical lessons learned during the pandemic, and strategic recommendations for building resilient and sustainable AMS practices. These outcomes highlight the importance of embedding AMS in emergency preparedness, strengthening diagnostic capacity, and ensuring evidence-based policymaking to combat AMR in future health crises.

**Conclusions:**

The COVID-19 pandemic highlighted the critical role of resilient and adaptable AMS in public health emergencies. Key lessons—such as integrating AMS into emergency planning, enhancing diagnostic capacity, and reinforcing infection prevention—must inform routine practice and preparedness strategies. Translating these insights into policy is essential to sustain AMS gains, guide AMR strategies, and ensure long-term protection of antibiotic effectiveness at national and global levels.
